# Martha Durkee

**Published:** 1899-12

**Authors:** 


MARTHA DURKEE, wife of Dr. Roswell Park, of Buffalo, died at Lebanon, N. H., November 14, 1899. Mrs. Park went east some weeks before on a visit and was ill about three weeks, though her condition was not considered serious until two days before her death, when alarming symptoms supervened, whereupon Dr. Park repaired to her bedside at once. Her sister, Mrs. Martin, of Chicago, was also with her. The remains were brought to Buffalo and the funeral was held from the residence of Dr. Park, 510 Delaware Avenue, November 16th.

Mrs. Park was prominent in church, charity and social circles and will be sadly missed from a home environed with refinement and happiness. Dr. Park receives the sympathies of wide professional and social circles in his bereavement.

